# Leaking Abdominal Aortic Aneurysm on Anticoagulants–Thromboelastography Assisted Management

**Published:** 2009-06

**Authors:** Anjeleena Kr Gupta, K K Narani, Jayashree Sood

**Affiliations:** Consultant, Deptt of Anaesthesiology, Pain & Perioperative Medicine, Sir Ganga Ram Hospital, Rajindra Nagar, New Delhi 110060; Sr Consultant, Deptt of Anaesthesiology, Pain & Perioperative Medicine, Sir Ganga Ram Hospital, Rajindra Nagar, New Delhi 110060; Sr Consultant & Chairperson, Deptt of Anaesthesiology, Pain & Perioperative Medicine, Sir Ganga Ram Hospital, Rajindra Nagar, New Delhi 110060

**Keywords:** Ruptured abdominal aortic aneurysm, Thromboelastography, Anticoagulant, Warfarin sodium

## Abstract

**Summary:**

Rupture of an abdominal aortic aneurysm (AAA) is a lethal event associated with a high mortality rate. In addition, the risk is compounded if the patient is on anticoagulants. Due to the recent advance in anaesthetic, operative and postoperative care, the patient if recovers from their emergency repair has a good long term survival. We describe the anaesthetic management of an elderly male on anticoagulant therapy presenting with ruptured AAA.

## Introduction

Abdominal aortic aneurysms (AAAs) represent a degenerative process of the abdominal aorta often attributed to atherosclerosis. Degenerative diseases account for more than 90% of all infrarenal AAAs. Risk factors include elderly age, male sex, Africoamerican race, increased height, weight, body mass index, body surface area, smoking, hypertension and coronary artery disease. In general, AAAs gradually enlarge (0.2-0.8 mm/ yr)^1^ and eventually rupture. The decision to operate depends on the size of aneurysm and associated comorbidities. For patients at higher risk viz hypertension, coronary artery disease, continued smoking or chronic obstructive pulmonary disease, the threshold for repair may be a diameter of 6-7 cm depending on their condition.^2^ Once this diameter is reached, risk of rupture increases with age. The perioperative management of patients undergoing surgery for aortic aneurysm repair depends on the coexisting morbidities along with the physiological changes occurring during aortic cross clamping and unclamping. We describe a case of eighty one year old male with known AAA who had denied operation in the past, presenting with rupture of AAA. Patient had a previous coronary artery bypass graft (CABG) with aortic valve replacement (AVR) for which he was on oral anticoagulant medication. This added to the complexity of the anaesthetic management.

## Case Report

The patient was an eighty one year old man weighing 60 kg with multiple comorbidities (bronchial asthma, coronary artery disease, post coronary artery bypass surgery(CABG) with aortic value replacement(AVR) 3 years ago) and a known AAA, admitted with abdominal pain for the last 4 days and syncopal attack for a day. The patient had refused surgical repair in the past. Patient was receiving regular treatment for asthma and oral anticoagulants warfarin sodium (2mg) and aspirin (75mg) once a day in view of AVR. He was conscious, oriented with normal vital signs (pulse rate 100/min, blood pressure 120/84 mmHg), good physical status and a positive attitude towards life. Abdominal examination revealed a soft, diffuse, pulsatile mass (12 × 8 cm) felt in left lumbar, umbilical, iliac fossa with associated abdominal tenderness. All routine investigations were normal apart from a raised serum creatinine (1.7 mg/dl) and deranged coagulation profile [international normalized ratio (INR) 3.8].

**Fig 1:**
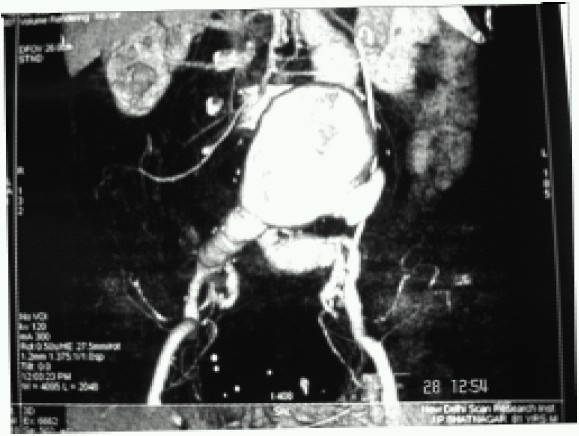
Spiral computed tomography showing an infrarenal aortic aneurysm with a contained leak (represented by arrow)

**Fig 2:**
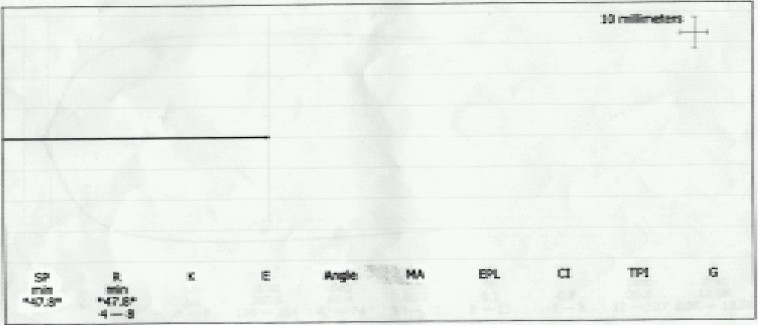
Preoperative TEG demonstrating increased reaction (R) time

**Fig 3:**
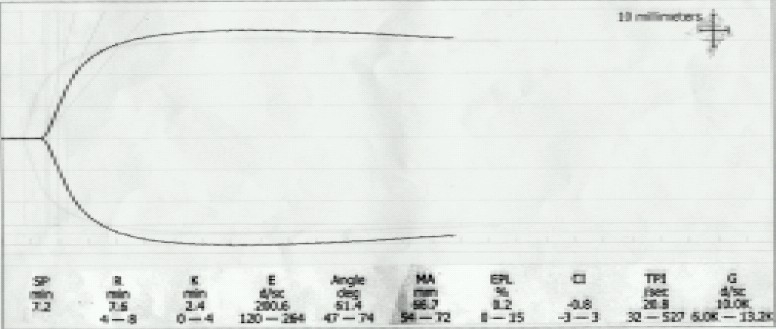
Pre-induction TEG demonstrating normal reaction time(R) and maximum amplitude(MA)

**Fig 4:**
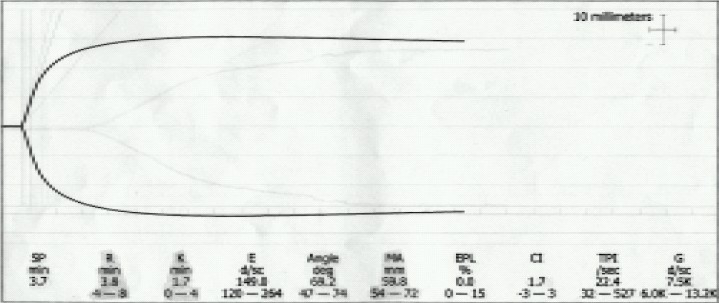
Prior to aortic clamp release, TEG demonstrating decreased reaction time and normal maximum amplitude

Patient had received warfarin sodium 2 mg on the day of surgery and was NBM since last 4 hours. Spiral computed tomography showed an infrarenal aortic aneurysm with a contained leak (Fig 1). So, patient was planned for an emergency surgical repair under general anaesthesia. Patient received intravenous ranitidine hydrochloride (50mg) and metoclopramide (10mg) 30 minutes prior to surgery. Patient was brought to the operating room wherein standard monitoring was instituted and a thrombelastography (TEG)^3^ by Thrombelastograph Analyzer (Haemoscope Corporation, Skokie IL, USA) was performed, in view of deranged coagulation, demonstrating an increased reaction (R) time which indicated the need to transfuse FFP (Fig 2). Two large bore intravenous access were secured and 100% oxygen was given by mask. Plan was to correct coagulation profile as the vitals were maintained. 10 units of fresh frozen plasma (FFP) were transfused and a repeat TEG was performed which showed a trend towards normalization with R time 7.6 min (normal 4-8 min) and maximum amplitude(MA) 66.7 mm (normal 54-72 mm) (Fig 3). Right internal jugular vein was cannulated with 7.0 FG triple lumen line and right radial artery was cannulated by Seldinger technique. Time taken for optimization of the patient was about 300 minutes following which general anaesthesia was instituted. Rapid sequence induction was performed with midazolam (1mg), incremental titrated doses of fentanyl (upto 300mcg), oxygen, nitrous oxide and isoflurane. Haemodynamic responses were blunted by intravenous preservative free lidocaine (1 mg/kg) and metoprolol on induction. To avoid the risk of rupture, intraoperative blood pressure was controlled (110-140 mmHg systolic) by intravenous glyceryltrinitrate infusion. Neuromuscular blockade using atracurium besylate (0.5 mg/kg) was given after the surgical incision was made. Anaesthesia was maintained by isoflurane in 60% nitrous oxide and analgesia provided by fentanyl boluses to maintain heart rate between 86–100/min. Patient was monitored using electrocardiogram (ECG), ST segment analyzer, pulse oximeter, capnometer, central venous pressure, arterial blood pressure, temperature and urine output. Core body temperature was maintained by convective warmer (Equator™, Smiths, Level 1, USA) and warm intravenous fluids using HOTLINE™ (SIMS, Level 1, USA). 500 ml normal saline was infused prior to aortic clamping and central venous pressure maintained between 7-11 mmHg. Intraoperatively, a 9×10 cm infrarenal aortic aneurysm was present along with thrombus in the aneurysm. 100-150 ml free intraperitoneal blood and a retroperitoneal haematoma was present. A Y shaped aortic PTFE graft was sutured. The straight portion of the graft was anastomosed to the distal aorta above the common iliacs while both the limbs of the Y were anastomosed to the right and left iliac arteries. Before aortic crossclamping 20% mannitol (1 gm/kg) and intravenous frusemide (20 mg) were given to maintain urine output around 125 to 250 ml/h. Minimal (250-300 ml) saline was given during aortic clamping. During the procedure aorta was clamped for 2 hrs. Intraoperative metabolic acidosis (base deficit – 7.0) was corrected by intravenous 50ml sodiumbicarbonate and another 50 ml was given just prior to release of the aortic clamp. Base excess at the end of the procedure was 1.8. Intraoperative blood loss was about 1800 ml which was replaced by six units of whole blood and 3 litres normal saline. Coagulation correction was done by further 4 units of FFP and one platelet apheresis. Prior to release of the distal clamp another TEG was performed that showed R time 3.8 min and MA 59.8 mm (Fig 4) indicating acceptable function of coagulation factors and platelets. Surgery lasted for 3 hours following which patient was electively ventilated in intensive care unit (ICU).

In ICU, patient was sedated and paralyzed with fentanyl and atracurium respectively for elective volume controlled pressure support (10 cm H_2_O) ventilation. He was extubated after 12 hours in the postoperative period. Blood pressure was controlled by glyceryltrinitrate infusion which was discontinued on second postoperative day. Patient recieved 3 units blood and FFP in the postoperative period and 100 ml 20% albumin daily to correct hypoproteinaemia. Low molecular weight heparin was started on the first postoperative day and subsequently, aspirin and clopidogrel were added to maintain anticoagulation. Patient developed icterus on the fourth postoperative day which resolved on its own on nineth day. The patient showed a remarkable recovery and was later discharged home on fifteenth postoperative day.

## Discussion

First AAA was described by Vesalius in the 16^th^ Century.^1^ Aneurysm is defined as a focal dilatation with a 50% increase over normal arterial diameter i.e. enlargement of atleast 3 cm of the abdominal aorta. Charles Dubost (1951) performed first AAA repair using a homograft which was later replaced by Dacron and Gore-Tex (i.e. polytetrafluoroethylene [PTFE]) fabrics, which retain tensile strength. In earlier days, even in elective cases the postoperative mortality rate was high (>25%) but today it ranges from 1.8–5% because of the improved surgical techniques and better perioperative management. Despite this improvement, the mortality is still high in cases of ruptured aneurysm even after they seek surgical intervention. In the presence of severe life threatening comorbidities including end stage lung disease or cardiac disease, the decision to operate is a do or die situation and one has to balance between risks and benefits. Although our patient was elderly with significant comorbidities, symptomatic with a contained leak and in addition was on anticoagulants, his overall physical status including a positive attitude towards surgery motivated us to accept the operation as an anaesthetic challenge.

Anticoagulants that patient had taken on the morning of surgery posed significant risk of bleeding during the surgical procedure. Ideally, the coumarins should be stopped 3-5 days before surgery. Spontaneous normalization of the INR takes about 4 days before surgery in patients with an INR between 2 and 3 who are taking warfarin. Vitamin K takes atleast 24 hrs to fully antagonize the effect but in urgent cases FFP or prothrombin complex concentrate is advocated.^4^ Aspirin affects the platelet function for the life of the platelet but several large studies have demonstrated the relative safety of central neural block along with antiplatelet therapy.^5^ In view of the contained leak decision was made to correct the coagulation profile as per the guidance of thrombelastograph.^3^ Right radial artery was cannulated for invasive arterial blood pressure monitoring and serial arterial blood gas analysis (ABG). Invasive monitoring could pose a risk of haematoma formation so, right internal jugular vein and right radial artery were cannulated after coagulation was corrected by FFP administration. Although central neural block could give the advantages of good intraoperative and postoperative analgesia but we decided to refrain from it for the risk of epidural haematoma.

Aim of anaesthesia was to have a haemodynamically stable, normothermic, pain free patient on completion of surgery. During induction and intubation a combination of fentanyl, midazolam and isoflurane was used along with preservative free lidocaine and metoprolol to minimise the CVS response and stimulation of the sympathetic system which could pose a risk of dissection of the aneurysm. Neuromuscular blockade was achieved after surgical incision to avoid sudden hypotension occurring with release of tamponade effect associated with muscular relaxation.

Intraoperative hypertension was avoided by providing adequate analgesia (IV fentanyl boluses) and glyceryl trinitrate infusion. Aortic cross clamping results in increased systemic vascular resistance leading to increased afterload which is associated with an increase in blood pressure by 7–10%.^6^ Diseased coronary system may be unable to respond to increased cardiac workload resulting in cardiac failure which could also occur secondary to overzealous fluid administration prior to cross clamping. The blood flow to the tissues below the clamp is dependent on the perfusion pressure rather than the preload and cardiac output. After infrarenal cross clamping, studies have shown 9–33% reduction in cardiac output.^7^ Our patient showed minimal response to cross clamping as is seen in patients with severe aortoocclusive disease who have well developed collateral circulation. We were able to maintain the mean arterial pressure (MAP) on higher side with crystalloids without ionotropic support. CVP was kept in normal range (7–11 mm Hg). Preoperative renal dysfunction has been suggested as a predictor for mortality in both ruptured and non-ruptured AAAs. The incidence of renal failure after AAA surgery is 5.4% of which 0.6% require haemodialysis. Infrarenal cross clamping reduces renal blood flow by upto 40% through the alteration of the renin angiotension system. In our patient, despite already deranged renal function adequate renal protection was instituted by 20% mannitol (1gm/kg) and frusemide in order to maintain urine output >125ml/hr. Since hyperglycemia^8^ and decrease in endtidal CO_2_ greater than 15% during aortic cross clamping^8^ are associated with a poor outcome, these parameters were monitored and kept within normal limits. During unclamping of aorta the CVP was kept on a higher side to avoid sudden fall in blood pressure^9^ and the clamp was gradually released to allow time for volume replacement and slow down the washout of the vasoactive cytokines, metabolites and cardiac depressant mediator from the ischaemic tissues.^6^ Intravenous sodium bicarbonate was administered to correct metabolic acidosis associated with the release of tissue metabolites.

Normothermia was maintained by forced air warming devices and fluid warmers in order to decrease the risk of perioperative myocardial ischaemia, dysrhythmias, coagulopathy and increased wound infection associated with perioperative hypothermia.

Postoperative analgesia was provided by IV fentanyl as thoracic epidural could not be placed with deranged coagulation because of the risk of epidural haematoma.^4^,^5^

Postoperative jaundice observed in the patient was attributed to multiple blood transfusion. It resolved on its own and the patient was discharged home after a suitable recovery.

Ruptured abdominal aortic aneurysm poses anaesthetic challenges due to the physiological changes associated with the disease process, aortic clampingunclamping, blood loss and major fluid shifts. These changes are further compounded by presence of anticoagulants. Effective management involved anticipation of these changes, vigilant monitoring and appropriate correction of anticoagulation by TEG guided FFP administration.
